# Antibody and Cell-Based Therapies against Virus-Induced Cancers in the Context of HIV/AIDS

**DOI:** 10.3390/pathogens13010014

**Published:** 2023-12-22

**Authors:** Julie Joseph, Grace Sandel, Ratuja Kulkarni, Reem Alatrash, Bobby Brooke Herrera, Pooja Jain

**Affiliations:** 1 Department of Microbiology & Immunology, Drexel University College of Medicine, Philadelphia, PA 19129, USA; jj932@drexel.edu (J.J.); ges59@drexel.edu (G.S.);; 2 Global Health Institute, Rutgers University, New Brunswick, NJ 08901, USA; ra996@scarletmail.rutgers.edu (R.A.); bherrera@globalhealth.rutgers.edu (B.B.H.); 3 Department of Medicine, Division of Allergy, Immunology and Infectious Diseases, Child Health Institute of New Jersey, Robert Wood Johnson Medical School, Rutgers University, New Brunswick, NJ 08901, USA

**Keywords:** viral infections, cancer, epitope vaccines

## Abstract

Infectious agents, notably viruses, can cause or increase the risk of cancer occurrences. These agents often disrupt normal cellular functions, promote uncontrolled proliferation and growth, and trigger chronic inflammation, leading to cancer. Approximately 20% of all cancer cases in humans are associated with an infectious pathogen. The International Agency for Research on Cancer (IARC) recognizes seven viruses as direct oncogenic agents, including Epstein–Barr Virus (EBV), Kaposi’s Sarcoma-associated herpesvirus (KSHV), human T-cell leukemia virus type-1 (HTLV-1), human papilloma virus (HPV), hepatitis C virus (HCV), hepatitis B virus (HBV), and human immunodeficiency virus type 1 (HIV-1). Most viruses linked to increased cancer risk are typically transmitted through contact with contaminated body fluids and high-risk behaviors. The risk of infection can be reduced through vaccinations and routine testing, as well as recognizing and addressing risky behaviors and staying informed about public health concerns. Numerous strategies are currently in pre-clinical phases or undergoing clinical trials for targeting cancers driven by viral infections. Herein, we provide an overview of risk factors associated with increased cancer incidence in people living with HIV (PLWH) as well as other chronic viral infections, and contributing factors such as aging, toxicity from ART, coinfections, and comorbidities. Furthermore, we highlight both antibody- and cell-based strategies directed against virus-induced cancers while also emphasizing approaches aimed at discovering cures or achieving complete remission for affected individuals.

## 1. Introduction

Historically, people living with HIV/AIDS (PLWHA) faced a heightened risk of developing cancer. In the early years of the HIV epidemic, specific types of cancer were frequently observed among this population, earning the label “AIDS-defining cancers”. These include non-Hodgkin’s lymphoma, Kaposi’s sarcoma, and invasive cervical cancer [1]. With the advent of antiretroviral medications, the life expectancy of PLWHA has substantially improved. Although PWLHA now face a reduced risk of HIV-related mortality, there is growing concern of other morbidities. Currently, 25% of all AIDS deaths stem from non-HIV-related causes, with cancer taking the lead. Among these, several are classified as AIDS-associated cancers, such as lung, anal, liver, colorectal, and testicular cancers. Their prevalence has surged among long-term PLWHA [2]. PLWHA are disproportionately exposed to factors that increase cancer risk, yet the precise mechanisms leading to cancer remain unclear. Factors like low white cell counts (<500)—indicative of a compromised immune system—as well as the effects of antiretroviral therapy (ART) can significantly impact cancer progression. Furthermore, coinfections with other cancer-causing viruses like HPV, Hepatitis, and EBV are more prevalent in PLWHA. Additional socio-economic factors, lifestyle choices, and behaviors, which are sometimes underestimated and overlooked, also contribute to the heightened cancer risk in PWLHA [3].

There is a growing urgency to better understand the mechanisms of cancer-causing viruses and to improve treatment options for individuals with chronic viral infections that progress to cancer. Approximately one in five cancers are attributed to infectious agents. The International Agency for Research on Cancer (IARC) identifies seven viruses as carcinogens, acting as direct oncogenic agents: the Epstein–Barr Virus (EBV), Kaposi’s Sarcoma-associated herpesvirus (KSHV), human T-cell leukemia virus type-1 (HTLV-1), human papilloma virus (HPV), hepatitis C virus (HCV), hepatitis B virus (HBV), and human immunodeficiency virus type 1 (HIV-1). More recently, Merkel cell polyomavirus (MCPyV) has also been linked to tumors, with ongoing studies exploring associations with yet-to-be-discovered tumors [4]. The determination of specific viruses that correspond to cancer etiology has had a profound impact on overall cancer intervention and treatment strategies. Furthermore, approximately 85% of virus-induced cancers are observed in developing countries, often grappling with public health crises due to limited resources and educational programs [5]. 

Viruses can initiate oncogenesis through various processes, including the introduction of oncogenes or altering the expression of pre-existing cellular genes. Some transforming viruses are replication-defective and may require a cellular cofactor to cause transformation. These include EBV and HHV-8. EBV, with its DNA genome, is associated with Burkitt’s lymphoma and some forms of Hodgkin’s disease. Other transforming viruses can cause cancer with their own viral genes. The most oncogenic virus identified by Zela and Gallo is HTLV-1, which can cause transformation without any cellular factors [6]. HTLV-1, an RNA virus, is the etiologic agent of adult T-cell leukemia/lymphoma (ATLL). Interestingly, HIV and HTLV-1, both members of the Retroviridae family, are known to infect similar cells with a tropism towards T cells. Both viruses have been shown to increase the frequency of CD4+ T cells, but with differing characteristics. 

DNA viruses like EBV and Simian virus 40 (SV40) exist in extrachromosomal episomes within the individual, establishing latency and reactivating lytic replication in response to cures, especially when the individual’s immune system is compromised. Some of these viruses are implicated in the development of single or multiple tumors [7]. HBV and HCV possess a double-stranded DNA genome or single-stranded RNA genome, respectively, and can infect hepatocytes and peripheral blood mononuclear cells, leading to chronic conditions like cirrhosis and subsequently hepatocellular carcinoma. HPV, a double-stranded DNA virus, is associated with cancers of the oropharynx, cervix, and anogenital tract. They induce transformation and establish a persistent infection with basal epithelial cells [6,8,9]. 

Certain cancers can serve as indicators of the progression of HIV to AIDS. Termed AIDS-defining cancers, they are common among individuals with compromised immune systems. Initially, common AIDS-defining cancers included aggressive B-cell lymphoma, invasive cervical cancer, and Kaposi’s sarcoma. As time has progressed, other cancers have been recognized as associated with PLWH. Non-Hodgkin’s lymphoma (NHL) was the primary AIDS-defining cancer in the US, and it is estimated to have a prevalence of 1194 cases per year in the US among PLWH [10,11,12]. Additionally, lymphomas are also included in the CDC’s list of AIDS-defining cancers, including Burkitt’s and immunoblastic lymphomas. These cancers are characterized by aggressive B-cell lymphomas, while some may have c-myc translocations. Many of the identified AIDS lymphomas are also EBV-infected tumor cells. 

**Figure 1:**
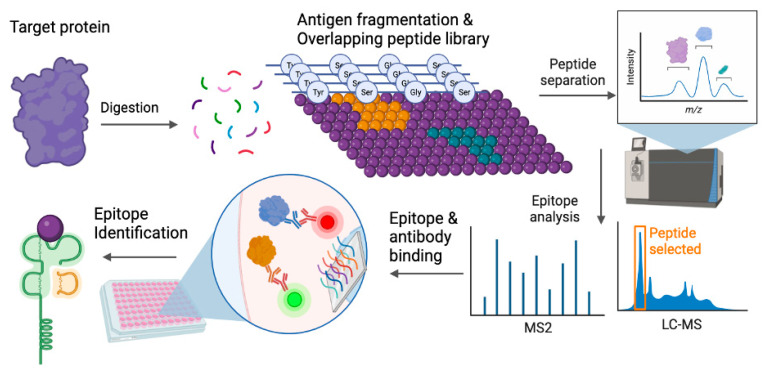
Epitope selection and identification. Target proteins of interest, often antigenic and immunogenic, are epitope-mapped to identify specific peptide sequences (purple dots representing various amino acid, orange and green represent peptide sequences that can elicit responses) that can elicit an immune response. The epitopes are identified by systematic screenings from mapped libraries and incorporate multiple conformations or binding domains. After mapping, candidate peptides are selected and screened for specific binding interactions between peptides and antibodies as well as affinity for MHC binding, leading to epitope identification. Illustration generated using BioRender ©2023.

The effects of these viruses on cancer development are highly complex. Interestingly, oncolytic viruses are emerging as a platform in immunotherapy treatments for patients with tumors, as they have the potential to bolster immune responses and efficiently target cancer cells. Key strategies to reduce the incidence of virus-induced cancers include infection prevention or eradicating the virus prior to cancer progression. Given that chronic infections are a hallmark of oncogenesis, targeting the pathogen before malignant progression represents a critical opportunity for cancer prevention. Several antiviral, interferon, and therapeutic vaccine approaches have been explored to target the virus before malignant transformation. One such strategy involves generating epitopes from viral antigens to prime or boost immune responses, either to lower viral burden, trigger latency, or facilitate clearance. The workflow for generating epitopes to improve antigen-specific responses is illustrated in Figure 1. This review discusses several immunoproteomic, cell-based, and antibody-mediated vaccine and immunotherapy approaches currently undergoing pre-clinical evaluation against cancer-causing viruses. 

## 2. Risk Factors Associated with Cancers in PLWH

Despite prevention measures and precautions, several risk factors can increase the likelihood of cancer in infected individuals. These factors can include sex, age, drug abuse, toxicity from ART, coinfections, and comorbidities.

### 2.1. Aging

One of the most significant risks for developing cancer, irrespective of source, is aging. The incidence of cancer among individuals above the age of 60 is approximately 1000 cases per 100,000 [13]. Several hallmarks of aging overlap with key contributors to cancer, such as genomic instability, epigenetic changes, accumulations of environmental and chemical pollutants, alcohol and tobacco usage, reduced sleep, and increased susceptibility to infections. Aging is also associated with immunosenescence and reduced immune function. This is often attributed to changes in immune cell phenotype and function, characterized by the dysregulation of surface markers, defects in cell signaling, and shifts in immune cell subpopulations [14]. Infection-related cancers are increasingly observed in individuals above the age of 50 living with HIV [15], HTLV [16], EBV [17], and Hepatitis B [18]. This is partially due to the chronic stimulation of the immune system, particularly by latent viral infections that periodically reactivate immune responses, exacerbating dysregulated immunity in the elderly. 

### 2.2. Drugs of Abuse and Opioids

Lifestyle choices also impact cancer incidence. Substance abuse represents an ongoing epidemic with significant physiological, psychological, and socio-economic consequences. According to the American Cancer Society, 30% of all cancer deaths, including about 80% of all lung cancer deaths, are linked to drug addiction [19]. It is well established that excessive alcohol consumption, tobacco use/cigarette smoking, and recreational drug abuse, particularly opioids, are major risk factors that heighten the incidence of several cancers due to their mutagenic effects. Furthermore, illicit opioid use has fueled outbreaks of several viruses, particularly HIV, HBV, and HCV. Previous studies have demonstrated that virus–drug interactions can accelerate viral pathogenesis, not only increasing transmission but also suppressing immune responses [20]. In longitudinal studies of people living with HIV, dangerous drug abuse was correlated with failure to maintain the viral load [21]. Many drugs of abuse also influence viral pathology and progression by altering the gut microbiome, affecting gut permeability, activating systemic immunity, and altering immune surveillance. Viral infections and drugs of abuse, when combined with cancer, can have devastating effects on prognosis, treatment strategies, remission, and survival outcomes. 

### 2.3. Soluble Factors, including HIV Proteins

Recently, several studies in both cancer and viral pathology have discovered roles for extracellular factors, such as exosomes and soluble receptor/ligands, in contributing to pathology as well as their utility as diagnostic biomarkers for disease severity. Often circulating in blood plasma or lymphatic fluids, these soluble factors have been correlated with viral load and reservoir persistence, as well as virus-specific immune function. One major soluble factor impacting pathology and immune responses is soluble immune checkpoint receptors. PLWH, both on and off ART, were shown to have elevated levels of sLAG-3, sPD-1, and sPD-L2 [22]. sCD27 in plasma is considered a biomarker for monitoring immune activation in PLWH receiving ART [23]. Furthermore, several of these immune checkpoints are also found on the surface of small extracellular vesicles, also known as exosomes, and can circulate systemically, eliciting responses in recipient cells [24,25]. These soluble immune receptors and exosomes have been shown to impact the efficiency of responses to cancer treatments [26]. Ongoing studies continue to explore their impact in the context of viral infections.

### 2.4. ART Toxicity

Antiviral drugs have significantly transformed the medical management of viral infections, aiding in prevention strategies against transmission. The FDA has approved two dozen ART drugs to combat HIV infection. The relationship between combination ART (cART) and cancer risk is complex and nuanced. Incidences of Kaposi’s Sarcoma and non-Hodgkin’s lymphoma (NHL) were elevated among HIV-positive individuals prior to cART, and with its use, there has been a decline. This is partially attributed to how cART improves immune function via viral suppression [27]. Recent randomized studies have indicated that cART reduces the risk of Kaposi’s sarcoma and NHL within the context of early infections before the development of overt immunosuppression [28]. As the associations between cART and cancer risk are based on malignancies categorized as AIDS-defining cancer, there is a disproportional bias that makes comparisons across other malignancies challenging. Non-nucleoside reverse transcriptase inhibitors have historically been associated with an increased risk of non-AIDS-defining cancers and Hodgkin’s lymphoma [29]. Evolving data from epidemiological surveillance have now established a framework to assess the interplay between HIV, coinfections, and cancer, though any implicit conclusions have yet to be determined. Recent experimental data have suggested that ART drugs may also have potential carcinogenic effects, increasing risks for cancer development. 

### 2.5. Comorbidities

Increased awareness of the gravity of developing chronic complications such as comorbidities and mental health disorders in people living with latent infections has emerged over the years. Various comorbidities such as stress; obesity; sleep deprivation; and cardiovascular, respiratory, and endocrinal diseases, including hypertension, COPD, and diabetes, can all impact the progression of cancer and viral infections. It is known that certain stages of cancer are affected by comorbidities, and the presence of a comorbidity increases the likelihood of being diagnosed with distant metastases, diverts early detection/diagnosis, and raises the chances of patients receiving an ambiguous stage of disease at diagnosis [30]. The role of ART in viral suppression has increased the survival rates of PLWH over the years. With more than seventy million people infected with HIV, about thirty-seven million are currently living with AIDS. However, a public health concern about HIV medicine is the risk of developing chronic morbidities [31,32]. Studies have provided evidence for the coexistence of HIV, cancer, and other non-communicable diseases. In their review, Gonah et al. mentioned that hypertension, which is among the most common cardiovascular diseases, diabetes mellitus, cancers such as breast and prostate cancer, and chronic respiratory diseases were prevalent among PLWH [33]. Coronary artery disease has contributed over the past two decades to deaths among PLWH. This is due to lifestyle choices, increased exposure to protease inhibitors through metabolic side effects, and increased inflammation [34]. Strong evidence also suggests that cancers such as lung, liver, and anal cancer are the leading causes of morbidity in PLWH. In the US, about one in every six deaths among PLWH was attributed to cancer from 2011 to 2015.

### 2.6. CoInfections

Coinfections play a significant role in increasing the incidence of cancer. Clinical observations and experimental studies have shown that a substantial number of individuals with cancer are coinfected with multiple pathogens, indicating a link between cancer etiology and progression. Infection-related cancer often varies substantially between various geographical regions, depending on the prevalence of infectious agents and racial/ethnic groups. As the probability of individuals being infected with two or more oncogenic agents is high, their role in cancer warrants extensive investigation. Some coinfections can be silent and not influence disease progression, while others can have severe implications for diagnosis, susceptibility, clinical presentation, and choice of drug regimen for treatment and prevention. As such, the NIH has launched grant programs to shed light on unestablished pathways in carcinogenesis that can inform prevention or treatment strategies for infection-related cancer. 

EBV and HTLV-1 are both lymphotropic viruses, with EBV favoring B cells and HTLV-1 showing tropism towards T cells. While singular infections with either virus are often asymptomatic, there are a few instances of acute lymphoproliferative disease or progressive leukemia as the chronic infection progresses [35]. Genetic factors, notably HLA haplotype, have been associated with the development of both EBV- and HTLV-1-associated leukemia/lymphoma. Interesting, carriers of the HLA-A2+ allele exhibit a lower frequency of EBV-associated NK/T-cell lymphomas, which is also the allele that dominates CTL responses against the immunodominant viral protein Tax in HTLV-1 [36,37,38]. Studies investigating the coinfection relationship have observed that EBV enhances HTLV-1 pathology, promoting more aggressive T-cell malignancies through the upregulation of adhesion molecules in an IL-4-dependent manner. EBV antigens, EBNA-1 and LMP-1, are positive in ATLL cells associated with skin malignancies. Studies have also suggested that HTLV-1 infection enhances EBV spread into endothelial and epithelial cells [35,39]. ATLL carries a very poor prognosis due to chemoresistance and significant functional immunosuppression. Throughout the clinical course of ATL, EBV coinfection has given rise to features such as lymphadenopathy; hepatosplenomegaly; skin, bone, gastrointestinal, and lung infiltrations; and hypercalcemia. Patients with HTLV-1/EBV coinfections often present aggressive ATLL [40]. Recently, the clinical dermatologic and histopathologic findings associated with cutaneous non-neoplastic and pre-neoplastic disorders with EBV and HTLV-1 coinfection were reviewed and updated to enhance diagnostic and treatment strategies [41]. Treatments for ATLL are often stratified by subtype - acute, lymphomatous, smoldering, or chronic. Chemotherapy is administered for aggressive-type ATLL (acute or lymphomatous), while ART has shown better outcomes for indolent-type ATLL, with a 100% survival rate [42]. First-generation polychemotherapy has been used against aggressive ATLL, with 16–36% achieving complete remission and sequential regimes of polychemotherapy yielding better outcomes. Aside from chemotherapy, allogenic stem cell transplantation is a curative treatment in aggressive-type ATLL [43,44]. Table 1 lists various current and potential treatment strategies for virus-specific cancers, which are discussed in detail here.

## 3. Cell-Based Vaccine/Immunotherapy Strategies

In tumor cells, the presence of viral gene products plays a crucial role in sustaining uncontrolled growth and immune evasion. This distinct characteristic sets tumor cells apart, making them prime targets for specialized treatments compared to conventional chemotherapy and radiation therapies.

### 3.1. Myeloid-Cell-Based Vaccine Strategies

**Figure 2:**
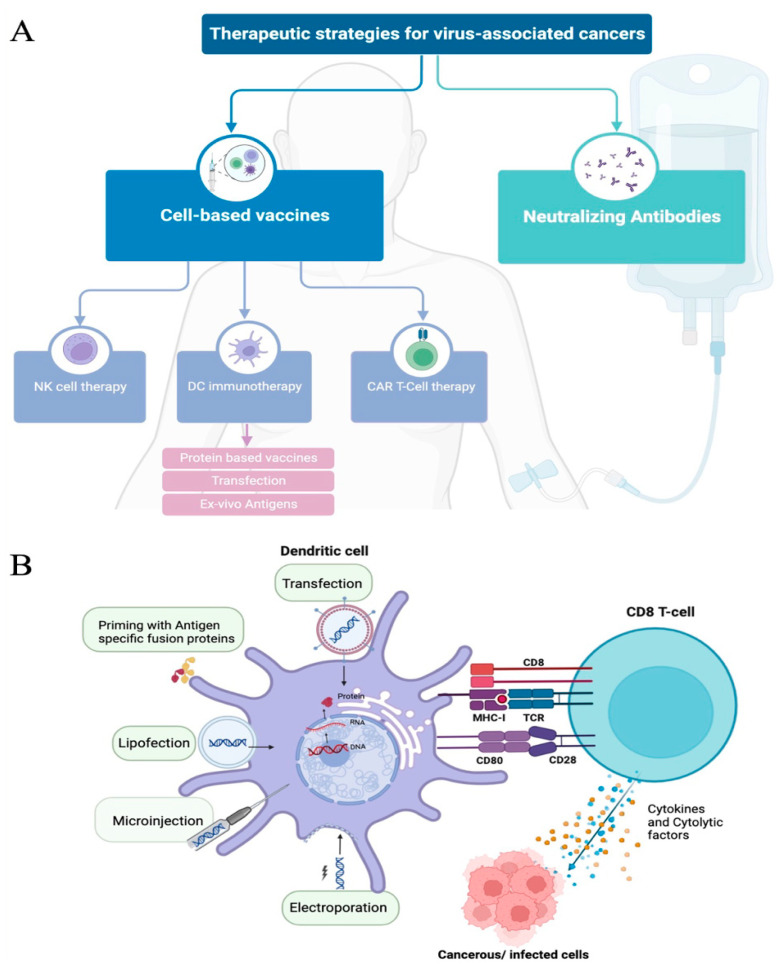
Therapeutic strategies for virus-associated cancers. (**A**) Flow diagram representing the current therapeutic strategies used and/or in development. Neutralizing antibodies and cell-based vaccines are the two major approaches. Cell-based vaccines target multiple cell types that play an important role in specific immune responses against cancer, with multiple priming strategies and modifications being used to improve the cell’s capacity to kill tumor cells. NK—natural killer cells; DC—dendritic cells; CAR—chimeric antigen receptor. (**B**) Dendritic cell priming strategies to improve targeted antigen-specific response. There are several strategies to enhance or prime dendritic-cell-mediated immune activations. The most common strategies are transfection and antigen pulsing. Other approaches, such as electroporation, lipofection, and microinjection, are also seen to prime DCs, inducing antigen-specific responses. Through these strategies, DCs can process and present antigens to MHC molecules and initiate specific cytolytic T-cell responses (releasing cytokines and chemokines (blue dots) or cytolytic factors (orange dots) that can target cancerous and/or infected cells. Illustration generated using BioRender © 2023.

Many vaccine candidates are currently in the stages of research and development (R&D), design, and manufacturing. These vaccines are engineered with the aim of delivering antigenic peptides to antigen presenting cells (APC). This triggers an immune response, ultimately leading to therapeutic benefit. Among APCs, dendritic cells (DCs) stand out as the most ideal candidates for anti-tumor and infectious disease vaccines. DCs surpass B cells and monocytes by a factor of 100 in their ability to prime antigen-specific cytotoxic T cell (AS-CTL) responses and sensitizing naïve CD4^+^ T cells. Additionally, DCs play a crucial role in establishing immunologic memory as they can also sensitize cells using soluble proteins [54,55]. Most antiviral DC vaccine strategies use viral peptides to stimulate autologous DCs, generating specific CTL responses. The prevailing approach in utilizing DC-based vaccines revolves around autologous therapies. This process initiates with the collection and isolation of monocytes from PBMCs, followed by treatment with growth factors (GM-CSF, IL-4) to induce differentiation into immature DCs. Throughout the maturation process, the antigen of interest is introduced, giving rise to monocyte-derived DCs (MDDC) [56]. These cells can then be adoptively transferred in vivo to initiate T-cell proliferation. This approach allows for the utilization of various antigens—antigenic peptides, inactivated whole viruses, glycoproteins, DNA vectors—all of which can lead to mounting an effective immune response. An overview of different DC-based therapeutic strategies are illustrated in Figure 2. The initial forways into developing DC-based vaccines were rooted in cancer immunotherapy studies. With the latest advancements in the field, these approaches are now being considered for viral infections, especially those caused by HIV, HBV, HTLV-1, and EBV. 

#### 3.1.1. Protein Based Strategies

Clinical trials revealed that vaccination with autologous dendritic cells loaded with HCV-specific cytotoxic T cell epitopes prooved to be safe, but were unable to generate sustained responses or alter the outcome of the infection [57]. In general, robust and sustained CD4^+^ and CD8^+^ T-cell responses against pathogen-derived epitopes are correlated with recovery from infections. However, in cases of persistent or chronic infections, weaker responses often arise due to limited epitopes. For example, in HCV infection, the compromised maturation of HCV-infected DCs hampers their ability to effectively present antigens to prime naïve T cells [58]. In mice, not a single epitope, but the whole viral surface induces immunogenicity in a DCs pulsed with hepatitis C envelope proteins E1 and E2 as a vaccine [59]. Adjuvants, particularily lipopeptides, have shown promise in inducing effective T-cell and antibody responses. These adjuvants activate innate signaling cascades, often via TLRs, thereby overcoming impairments with antigenic peptide presentation. In HCV, DCs pre-treated with a bacterial toxin fused core antigen was found to be safe and able to prime HCV core antigen-specific CTLs in vivo [60]. Another strategy using a fusion protein incorporating the extra domain A (EDA) from fibronectin and the HCV NS3 led to strong and sustained NS3-specific CD4^+^ and CD8^+^ T-cell responses, along with the downregulation of intrahepatic expression of HCV-NS3 RNA [47]. Additional approaches observed in HCV included loading core antigens, as well as NS3 and NS4 [45], and utilizing NS5 protein-coated microparticles to elicit antigen-specific CTL activity in mice, resulting in the reduction of tumor growth in viral protein expressing cells [46].

The degree of lipopeptide-mediated DC maturation and the strength of subsequent immune responses suggest that, in comparison to the conventional DC maturation processes involving a cytokine cocktail in vitro, lipopeptide-based epitopes hold significant promise for DC-based immunotherapies. In the case of HIV-1, DC-based strategies feature chimeric monoclonal antibodies directed to DC surface receptors fused to an antigen of interest. This multiepitope immunogen-based strategy garnered stronger CD4^+^ and CD8^+^ T-cell responses when compared to DC-based vaccine strategies in mice [48]. Studies have also supported the use of higher animal models, such as Indian and Chinese rhesus macaques, where putative epitopes with associations to HLA-I alleles correlated with low viral set point and slow disease progression—HLA-B*501, and rapid viral set point and rapid disease progression—HLA-B*18 [61]. One of the first Phase I therapeutic vaccine trails in Uganda showed that a detoxified anthrax-derived polypeptide fused to the subtype C HIV gag protein p24, was well tolerated amongst thirty HIV positive volunteers. CD4 counts were significantly higher in vaccine recipients compared to placebo after 12 months. Initial results suggest that therapeutic immunization may be beneficial in select individuals to bolster immune responses [62]. 

At present, numerous clinical trials have showcased the safe and effective transfusion of EBV-specific cytotoxic T lymphocytes and other cell therapies for the prevention and treatment of diseases attributed to EBV [59,63,64]. With EBV, potential epitopes often derive from fragments of products from EBNA1 and LMP2 genes, commonly expressed in latent stages inside EBV-related carcinomas. While DCs have been successful in activating CD4^+^ and CD8^+^ T cells, the balance between MHC class I and II epitope targets from these genes remain unachieved [49,65]. With regards to HTLV-1, naturally presented Tax epitopes were seen to initiate antigen specific CTL response in vivo but failed with the depletion of DC, giving rise to the Tax (11–19) epitope as the current gold standard candidate for DC-based anti-HTLV-1 vaccine [50]. Almost all protein-based DC vaccine strategies remain in the pre-clinical phase.

#### 3.1.2. Transfection Strategies

Another commonly employed approach for DC-based epitope therapies involves transduction with recombinant AV (adenovirus) or AAV (adeno-associated virus), resulting in multiepitope CD4^+^ (Th1) and antigen-specific CTL responses. Transfection with lentiviral vectors also stimulates CD4^+^ and CD8^+^ responses while transfection with cytopathic RNA can lead to cross priming of AS-CTLs. In HCV, recombinant AVs expressing HCV-core protein, NS3 protein, have shown promise in developing safe, intrinsic adjuvant effects with heterologous antigen exposure to activating immune responses [66]. 

#### 3.1.3. Ex Vivo Antigen Strategies

Using HCV-like particles (HCV-LP) of the core protein and HCV-LP E2, receptor-mediated DC-HCV-LP interactions revealed uptake and DC activation, ultimately inducing HCV-core specific CD4^+^ and CD8^+^ T cells [67]. As the efficacy of immune therapy and therapeutic vaccines against HIV infection has been modest at best, it has been hypothesized that ex vivo generated DC therapeutic vaccines aimed to induce effective HIV-specific immune responses might overcome some of these challenges. However, despite these encouraging results, functional cure has not been reached with this strategy in any patient. 

#### 3.1.4. DC Immunotherapy Strategies

Recent advancements in DC-based vaccines for cancers have paved the way for their application in HIV-1 infection. DC-based vaccines have been assessed in clinical trials with HIV-1 patients, in which the virus is effectively suppressed by ART. The primary goal of these vaccines are to induce HIV-1 antigen-specific T-cell responses, with the hope that they could help clear or eliminate the need for long-term ART. More recent studies have investigated the use of patient-derived HIV-1 antigens and autologous Gag and accessory proteins Nef, Rev, and Vpr. The use of HIV-1 mRNA encoding the Tat and Vpx proteins has also been studied. However, meta-analysis studies estimate the success rate of recent HIV-1 DC-based immunotherapy trails to be no more than 38% [68]. Harnessing DCs to treat HIV is considered a key strategy for improving anti-HIV treatment and functional cures. Teodora ds Silva et. al., discuss how ongoing strategies for using a DC based immunotherapy can be improved, and highlight current challenges and ongoing trials [69]. 

#### 3.1.5. The Clinical Success of Myeloid-Cell-Based Therapies

With over 412 clinical trials in various stages (recruiting, ongoing, or completed), several phase trials for single and multiepitope DC-based vaccines for cancer patients have shown promising results. In a trial with glioblastoma patients, treatment involving autologous DCs pulsed with MHC class I peptides from tumor-associated antigens expressed on gliomas, particularly those overexpressed in their cancer stem population, demonstrated a positive correlation with prolonged overall survival and progression-free survival in newly diagnosed patients [51]. Vaccine trials that have evaluated the safety and feasibility of mature DCs pulsed with lysates prepared from HCC tumors showed it to be safe and tolerable. Additionally, patients who received 2-doses, initial and a booster after a 12-month period, showed a significant increase of 1-year overall survival. The evidence for the development of DC vaccine-based immunotherapy holds great promise, particularly in combination with immune/anti-cancer therapies for a personalized therapeutic approach to treating patients [70]. 

### 3.2. Lymphoid-Cell-Based Strategies

Generating vaccine induced protection against viral infections has proven challenging, particularly due to the lack of efficiency in CD8+ T-cell-based vaccine trails. Evidence, based on responses in challenged non-human primate (NHP) models, however does indicate that improved CTL mediated strategies could benefit HIV vaccines [71]. While eliciting broadly neutralizing antibodies (bNAbs) is considered the holy grail to vaccine research, the natural induction and development of antibodies against HIV can take years and can often be inefficient due to high levels of somatic mutations and INDELs. CD8+ T-cell directed vaccines can target more conserved and cross-reactive epitopes that have a fitness cost against mutations. 

#### NK Cell Therapy

In cancer tissue, NK cell counts are often lower than normal, which contributes to cell growth and dysregulation of NK function in immunity. Although crucial in innate immune defense against carcinogens, NK cells are functionally suppressed in the tumor microenvironment. Targeting these NK cells is considered a pathway in immunotherapy, as higher NK cell cytotoxicity is associated with a reduced cancer risk. NK cells play a pivotal role in promoting the maturation and activation of DCs, macrophages, and T cells via IFN-γ and chemokines. Notably, NK cells possess independent cytotoxicity, as they do not rely on MHC-antigen stimulation. In leukemia patients undergoing allogenic stem cell transplantation, NK cells are the first lymphocytes to appear [72]. While adoptive transfer of NK cells in cases of infectious disease complications are still being explored, evidence suggests their effectiveness against viruses and bacteria. However, determining the optimal timing for NK cell transfer to maximize benefit while minimizing harm remains a challenge [73]. Allogenic NK cell therapy encompasses both peripheral blood-derived (PB-NK) and umbilical cord-derived (UB-NK) therapies, both of which have shown promise in preclinical studies. However, each come with their respective limitations, such as poor efficacy of cryopreserved and weaker cytotoxicity. Generally, PB-NK and UB-NK adoptive cell therapies have demonstrated success in the treatment of hematologic cancers, though challenges like limited donor supply and resource constraints persist. Clonal NK-cell lines, such the NK-92 immortalized cell line derived from a lymphoma patient, have also been used in NK cell-based therapies. NK-92 exhibits significant potential in targeting metastatic tumor models, though it faces difficulties like limited in-vivo expansion and a lack of benefit for patients with refractory/relapsed AML. Another adoptive cell therapy, IPSC-derived NK cells, are easier to engineer and possess greater expansion potential. These cells have successfully delayed tumor progression in a xenograft ovarian cancer model [74]. In the context of HIV-1 infection, NK cells play a crucial role in regulating the antiviral response by secreting IFN- and MIP-1, and shaping the induction of antibodies through elimination of follicular T cells [75]. NK cells also produce chemokines that inhibit HIV-1 entry to CD4^+^ T cells and limit its spread. Additionally, NK cells are instrumental in preventing mother-to-child transmission of HIV-1 and have been associated with natural resistance to HIV-1 infection in HIV-1 exposed, sero-negative intra-venous drug users (HESN-IDUs) [75]. In HIV-infected individuals, there is an increased NK cell cytotoxicity in response due to HLA downregulation, and HIV induces upregulation of specific ligands for NKG2D receptor, enhancing NK cell-mediated killing. Several studies have demonstrated that HIV-specific antibodies mediate non-neutralizing functions apart from neutralization, and these non-neutralizing functions may be involved in protection against HIV infection. This includes enhancing cytokine/chemokine production and lysing of target cells by NK cells. Strategies to evade antibody-dependent cellular cytotoxicity (ADCC) have been developed, including the reduction of tetherin- a cellular host restriction factor, which sequesters viral particles and protects infected cells from the NK cell activity [76]. Long-term non-progressors, controllers, and HESN exhibit mechanisms of HIV resistance, including increased cytotoxicity and higher production of soluble mediators by NK cells. The presence of the activator receptor KIR3DS1 on NK cells in combination with HLA-Bw4-I80 is associated with delayed AIDS progression and resistance to infection [76]. IL-15 has been shown to boost NK cells activity and kill latently infected cells in presence of ART, resulting in a diminished viral reservoir size and viral eradication in infected individuals. Given the role of NK cells in the protection against HIV infection, targeting NK cells by immune check point modifications could potentially serve as a new therapeutic approach for HIV specific cancers. It was demonstrated that dual blockade of PD-1 and IL-10 in untreated and ART-suppressed PLWH, could enhance NK cell activity, restoring CD4+ T-cell function [52]. Primary EBV infections may lead to the development of infectious mononucleosis (IM). EBV strains that encode for specific LMP-1 peptide variants elicit strong NK and CD8^+^ T-cell responses, and the presence of these strains is associated with progression of EBV, along with post-transplantation lymphoproliferative disorders [77].

### 3.3. Chimeric Antigen Receptor T-Cell (CAR-T) Therapy

The application of chimeric antigen receptors (CARs) represents a promising strategy to enhance the precise targeting and killing of cells infected with HIV, EBV and HPV [78]. While CAR-T therapy has shown notable success in specific subsets of B-cell leukemia or lymphoma, it is not without its limitations. Those include potentially life-threatening toxicities, modest anti-tumor activity, the issue of antigen escape, restricted trafficking, and limited tumor infiltration. Moreover, its efficacy in treating solid tumors is hampered by the challenges CAR-T cells face in reaching and penetrating these types of tumors. Researchers are actively addressing this issue by exploring alternative delivery routes that would negate the need for CAR-T cells to navigate to disease sites [79]. 

CAR-expressing T cells have the potential to confer stable and durable immune surveillance to HIV reservoirs. The modification of T cells through CAR-T therapy hold substantial promise for the treatment of B-cell malignancies [80]. This approach involves the genetic modification of T-cells to target tumor-associated antigen. These cells can be sourced from the patient or a donor, then isolated and genetically engineered. Encouraging outcomes have emerged from CAR-T cell therapies in the treatment of hematologic malignancies and B cell lymphomas [81]. This therapy also presents an attractive option for combatting viral and fungal infections in immunocompromised individuals [82]. 

In the case of HIV, CAR-T cells can be tailored to specifically target HIV, particularly leveraging the potent anti-HIV broadly neutralizing antibodies (bNAbs). Anti-HIV CARs engineered on the bases of bNAbs has shown to actively kill HIV-infected cells. In this case, the gene expression cassette of the receptor showed this activity when recombined into the CCR5 locus, which might be a promising approach for a new HIV CAR-T-cell therapy. In fact, one potential CAR T-cell candidate therapy has made its way to clinical trials with two patients has received CAR T-cell targeting HIV envelope glycoprotein gp120 (LVgp120duoCAR-T) [53,83].

Extensive research has been devoted to enhancing CAR-T cell function and persistence, particularly in CARs targeting CD19 and other tumor-specific antigens. To address challenges related to engraftment, function, and persistence, CD4-CAR T cells in an HSPC-based approach have demonstrated successful T-cell differentiation and significant suppression of HIV replication in humanized bone-marrow-thymus-live mice. The CAR structure comprises three key domains: extracellular, transmembrane and endo-domain. For HIV patients, CD8^+^ T cells are transduced with CAR genes and subsequently reinfused following in vitro confirmation of their anti-HIV specificity [84]. Furthermore, the utilization of dual-antigen receptor T cells derived from IPSCs offers a strategic means to prevent tumor antigen escape. These cells are engineered to express a CAR targeting the LMPi and a T-cell receptor specific to LMP2, demonstrating promise in treating EBV-associated lymphomas [85].

## 4. Neutralizing Monoclonal Antibody-Based Immunotherapies

Neutralizing monoclonal antibody-based immunotherapies are a type of treatment that involves the use of monoclonal antibodies to target and neutralize specific molecules or pathogens. Monoclonal antibodies are laboratory-made molecules designed to mimic the immune system’s ability to fight off harmful pathogens such as viruses or cancer cells. In the context of infectious diseases, including viral infections, neutralizing monoclonal antibodies are engineered to recognize and bind to specific viral proteins, preventing the virus from entering or infecting host cells. This interference with the virus’s ability to replicate and spread can be a valuable strategy in treating or preventing viral infections. In the context of cancer, monoclonal antibodies can be designed to target specific proteins on the surface of cancer cells. By binding to these proteins, the antibodies can interfere with the growth and survival of cancer cells. Monoclonal antibodies have been highly successful during the past decades for therapy of many diseases, primarily cancers and immune disorders. They are relatively safe, especially human mAbs that have evolved in humans at high concentrations to fight diseases and long-term use may not lead to toxicities [86]. In non-Hodgkin lymphoma (NHLs), Brentuximabvedotin (Adcetris), Ibritumomabtiuxetan (Zevalin) and rituximab (Rituxan), has been used for treatment of NHLs and showed promising results [87]. nAb therapy has been studied in several cancers including virus-associated cancers [88]. Despite the success of monoclonal antibody (mAb) therapy in treating cancer, the significant challenge of clinical resistance to these agents persists. A minority of patients exhibit a positive response, while the majority develop resistant disease within a year [89]. An inherent limitation of mAb therapy lies in its efficacy being contingent on the expression of target molecules by tumor cells that can be bound by the antibodies [90]. The emergence of mutations in the antibody target and related downstream signaling molecules can result in acquired resistance to mAb therapy, activating alternative pathways for growth or survival signaling [91].

### 4.1. Neutralizing-Monoclonal-Antibody-Based Immunotherapies across Pathologies

Neutralizing antibodies constitute a well-esatblished approach for combating viral diseases. The overarching goal of nAb therapy is to neutralize target cells and label them for elimination. By leveraging a suite of bioinformatic tools for sequence analysis, epitope mapping, microarrays, and high-throughout immunoassays, researchers can engineer epitope-based vaccines with potent passive immunity potential, minimizing the undesired side effects associated with conventional vaccination. These nAbs exhibit the capacity to to respond to a broad spectrum of viral pathogens, enhacing their utlity across a range of infection scenarios [92]. Given that a majority of monoclonal antibodies fall under the category of broadly neutralizing antibodies (bnAbs), they possess a range of neutralization capabilities [93]. These capabilities hinge on the computational design of the monoclonal antibody, as well as their efficiency of binding. Ideally, effective nAbs should bind with high affinity to an target epitope on the viral particle.The specific epitopes of interest vary depending on the virus in question, and different epitopes may be used to target a particular virus.

monoclonalAbs are also employed in cancer therapies that include antibody drug conjugates (ADCs) and bispecific monoclonal antibodies (bsAbs). ADCs contain a monoclonal antibody and a linked cytotoxin that gets released into the cytoplasm of the tumor cell when the monoclonal antibody binds and recognizes the target cell [94]. This cascade triggers downstream effects, including DNA denaturation, cell division inhibition, and cytotoxicity of tumor cells. Bispecific antibodies are distinctive in that they contain two antigen binding sites on the same molecule [95]. There are three different classes of bispecific antibodies, cytotoxic effector cell redirectors, tumor-targeted immunomodulators, and dual immunomodulators [96]. Moreover, every bispecific antibody follows one of two general design formats, either a single-chain variable fragment (scFv)-based antibody or the full-length IgG-like asymmetric antibody [97]. Both design types enhanced the killing capacity and anti-tumor efficacy of the treatment [94]. Table 2 provides a list of available bnAbs at various stages of development for different viral infections.

### 4.2. HIV-1 bnAbs as a Response to the High Risk of Cancer

Efforts in HIV vaccination have shifted towards the development of various bnAbs therapies. Multiple binding sites have been investigated, including the HIV-1 V3 loop, CD4+ binding site, V1V2 apex, gp120-gp41 interface, “silent face” of gp120, and membrane proximal external region (MPER) [98]. Sequential immunogen design strategies, such as B-cell lineage immunogen design, germline targeting, mutation-guided immunogen design, and structure-based immunogen design, have been exploited to develop an effective bnAb for HIV [99]. These strategies have created the most successful non-vaccine HIV-1 treatments to date [100]. Among these triumphs, VRC01 has been shown to be safe and well-tolerated in phase I studies and for multiple HIV-1 escape mutants [100,101]. VRC01 targets the HIV-1 CD4^+^ binding site and proves able to neutralize HIV strains, capture virions, and facilitate ADCC [102]. Structurally, VRC01 complexes with the HIV-1 gp120 core, mimicking the interaction between CD4 and gp120 in virally infected cells [103]. Specifically, VRC01 contacts the gp120 loop D, along a five amino acid motif of 278T(D/N) NAK283, and the V5 region of gp120, near residues 458 to 467, to maximize neutralization potential [103]. The target epitopes of interest, especially the CD4^+^ binding site, are relatively conserved, but can have slight variation, causing the ability for CD4 escape from bnAbs [101]. Most escape mutants, however, have maintained or gained greater sensitivity to VCR01 [101]. Another neutralizing antibody, PGT121, targets the glycan-rich V3 of the HIV-1 envelope glycoprotein, gp120 [98]. PGT121 is versatile in its clinical use. For example, this neutralizing antibody can be administered in conjunction with complimentary neutralizing antibodies, such as VRC07 and PGDM1400, to combat resistance [98]. Additionally, bispecific antibodies are being proposed for a more potent neutralization response, including the combination of PGT121 x VRC01 [104].

**Table 2:** Known antibodies and epitopes for viruses and virus-specific cancers.

Antibody	Infection	Cancer	Epitope Sequence	Protein Target
***Non-cancer-causing viruses***				
VRC01	HIV-1		278-T(D/N) NAK-283 of loop D residues 458 to 467 of V5 region.	gp120; mimics CD4 binding [91]
PGT121			V3 loop	gp120 [105]
***Cancer-causing viruses***				
3A3	EBV	NHL	complex with DII and DIV	gB [106]
3A5			complex with DII and DIV	gB [106]
1D8			discontinuous epitope involving DI/DII binding domain	gHgL [106]
AMM01			interface with DI/DII	gHgL [106]
VIR-3434	HBV/HDV	HCC	antigenic loop of HBsAg	HBsAg [107]
PC151-1, HEPC122, HEPC154, HEPC153	HCV		AR3	E1/E2glycoprotein complex [108]
HEPC111, HEPC130			AR4–5	E1/E2 glycoprotein complex [108]
Hu-LAT-27	HTLV-1	ATL	L-P-H-S-N-L	gp46; rat LAT-27 CDR with human IgG1 backbone [109]
***Cancer associated with AIDS***				
Brentuximab Vedotin	HIV-1	NHL	TNFRSF8	CD30 [110]
Glofitimab		Diffuse Large B Cell Lymphoma (DLBCL)	72IPAGIYAPI80 146FLKMESLNFIRAHTPYINIYNC167 on CD20	monovalency for CD3 protein; bivalency for CD20 protein [111]
Epcoritimab				CD3 and CD20 [112]
Cadonilimab		Cervical Cancer		CD3 and CD20 [112]
Tisotumab Vedotin				TF-011 [113]

### 4.3. HTLV-1 bnAbs for Virus-Associated Cancer

Monoclonal antibody vaccines are also being considered for HTLV-1. Several epitopes, from both B and T cells, have been identified for potential multi-epitope vaccine development against HTLV-1. Of these epitopes, KEADDNDHEPQISPGGLEPPSEKHFR and DGTPMISGPCPKDGQPS, spanning from 324-349 and 252-268, are the most promising for B cells, being both non-toxic and located on the exterior of the protein [114]. Likewise, LLFGYPVYV, ITWPLLPHV and GLLPFHSTL, ranging from 11–19, 163–171 and 233–241, are the most antigenic among CTLs [114]. Knowledge of both B and T cell epitopes will be instrumental in targeting Tax, one of first proteins produced in a cell infected with HTLV-1. Hu-LAT-27 is a monoclonal antibody derived from rats that orechestrates the neutralization of viral particles in HTLV-1 patients [115]. Engineered using Chinese hamster ovary (CHO) cells, this antibody targets the gp46 glycoprotein of the HTLV-1 enveloped virus [116]. Hu-LAT-27 comprises a complementariy-determinina region (CDR) that combines rat LAT-27 and a human IgG1 backbone [116]. CHO cells, known fro their widespread application in biopharmaceuticals, boast the ability to yield abundant cells rapidly, useful for mass producing neutralizing antibodies [117]. The neutralization epitope of Hu-LAT-27, found within amino acid 191–196 (Leu-Pro-His-Ser-Asn-Leu), exhibits a a wide spectrum of reactivity [115].

### 4.4. Epstein–Barr Virus: Broad Prevention of Infection

In the quest to thwart EBV infection, several epitopes have piqued interest. Notably, gp350 and gp220 are two potential therapeutic antigens, alongside gB, gHgL, and gp42 [118,119]. While gp350 is currently under investigation in clinical studies, the remaining glycoproteins are undergoing pre-clinical scrutiny [119]. Both gp350 and gp220 form an attachment protein, known as gp350/220 [119]. Each of these glycoproteins play a role in viral entry during EBV infection [119]. Specifically, glycoprotein B(gB) is the primary fusogen essential for EBV entry into host cells, particularly B cells and epithelial cells [106]. There are three known monoclonal antibodies that target the gp350 protein, 72A1, C1, and HB5 [119]. Additinally, monoclonal antibodies, 3A3 and 3A5, which target glycoprotein gB at domains D-II and D-IV, respectively, have been shown to inhibit membrane fusion of EBV with its host cell [106].

Another glycoprotein of interest is the glycoprotein H glycoprotein L heterodimer (gHgL). GHgL is normally important for membrane fusion [106]. Specifically, the DI/DII linker regions of gHgL are involved in gB binding and activation for epithelial and B cell entry [106]. Multiple antibodies exist that target gHgL, including E1D1, CL59, CL40, AMMO1 and 1D8 [106]. Of these antibodies, 1D8 and AMMO1 have exhibited potent binding and neutralizing capacity [120]. 1D8 and AMMO1 both target gHgL, but at slightly different complementarity determining regions of the gHgL DI/DII domains [120]. Both 1D8 and AMMO1 have the capacity to prevent infection in both epithelial and B cells, making them more enticing as a therapeutic option than their counterparts, like CL59 and CL40, who only prevent infection in epithelial cells [121]. 

### 4.5. Hepatitis bNabs

Mutations can confer resistance to many broadly neutralizing antibodies [122]. Mutations, like escape substitutions, will modify the target epitope or interfere with binding through allosteric effects. The optimal function of a broadly neutralizing antibody then becomes dependent on the severity of these mutations [123]. Specifically concerning HBV, the Hepatitis B surface antigen (HbsAg) tends to be a target for therapeutic treatment of HBV. However, mutations are known to occur, especially in the preS1, pre-S2, and “a” determinant regions of the antigen [124]. VIR-3434, an antibody treatment for HBV, targets a conserved epitope on the antigenic loop of HbsAg [125]. VIR-3434 is a promising treatment that is in clinical development for patients with Hepatitis B and D, and neutralizes both HBV and HDV in the blood [126]. For Hepatitis C, broadly neutralizing antibodies are being uncovered, like PC153, HEPC151-1, HEPC154, HEPC122, HEPC111, HEPC130, and HEPC108, that all target the E1/E2 envelope complex at various binding residues [108]. It appears that some bnAbs may have significant binding overlap with the portion of E2 that interacts with CD81 [122]. This evidence raises the possibility for an HCV vaccine development [122].

### 4.6. bNabs in HIV-Associated Cancers

As of June 2022, there are 12 FDA approved conjugate drug therapies available for treatment of various cancers. These treatments include Brentuximab Vedotin targets AIDS-related cancers. Brentuximab Vedotin (BV) is an ADC therapy that combines mouse human chimeric IgG1 anti-CD30 mAb (cAC10) to synthetic dolastatin 10 analog monomethylauristatin A (MMAE) using a dipeptide linker [127]. BV acts by binding to the CD30 protein, primarily located on B and T cells, that plays a role in Th1 and Th2 responses [127]. CD30 is a transmembrane protein (TNFRSF8) that belongs in the Tumor Necrosis Factor (TNF) family and is abnormally expressed in tumor cells [128]. Once BV binds and is brought into an infected cell, MMAE is released and disrupts microtubule development, causing cell cycle arrest and apoptosis [128]. Brentuximab Vedotin is currently marketed for Hodgkin lymphoma (HL) and non-Hodgkin lymphoma (NHL) treatment [127]. To treat these cancers, combination therapy can be beneficial. Combination therapy is achieved for patients with HL by combining Brentuximad Vedotin with doxorubicin hydrochloride (Adriamycin), bleomycin sulfate, vinblastine sulfate, and dacarbazine (AVD) therapy [128]. 

Bispecificity refers to an antibodies ability to bind to two target epitopes. Their functionalities include interferences with multiple processes, bringing targets close to where effector functions may take place, or amplification of immune responses due to increased availability of binding sites. Glofitamab and Epcoritimab are both bispecific antibodies targeted for Diffuse Large B Cell Lymphoma (DLBL) treatment [97]. Glofitamab exhibits monovalency for CD3 targeting protein and bivalency for CD20 [112]. The bivalency of Glofitamab’s CD20 targeting regions increase binding potency and make them marketable for replacement of other anti-CD20 antibodies in the future [129]. Glofitamab also shares the same epitope targeting sequences as another anti-CD20 antibody, called Obinutuzumab [112]. Epcoritimab, like Glofitamab, is used in treatment of DLBL. Epcoritimab is also a CD3xCD20 bispecific antibody that directs targeted B cell CD20 killing [130].

### 4.7. HIV and HPV Infections in Cervical Cancer

Bispecific antibodies and ADCs have applications in other AIDs-defining cancers, like cervical cancer. Cadonilimab is a bispecific antibody therapy primarily used for treatment of cervical cancer. The antibody features both anti-CD4 and anti-CTLA4 domains [94]. Similarly, Tisotumab vedotin (Tivdak) is an ADC composed of human IgG1ҡ antibody targeting tissue factor expressed on the surface of cancer cells—TF-011, monomethyl auristatin E (MMAE), and a peptide linker [131]. Tivdak was also recently approved for treatment of recurrent or metastatic cervical cancer with disease progression on or after chemotherapy. The binding of MMAE to tubulin disrupts the microtuble network of actively dividing cells resulting in cell cycle arrest and apoptotic dealth of tumor cells. Tisotumab vedotin is seen to mediate ADCC and anti-body dependant cellular phagocytosis [132].

### 4.8. Alternate HIV-1 Treatment Strategy

Recent developments in HIV-1 treatment strategies have aimed at using a single molecule that can both activate and eliminate HIV-1 reservoir cells to manifest a functional cure for HIV/AIDS. There was careful consideration to not cause cytokine release syndrome (CRS) and associated adverse effects (AE). The trispecfic N6/αCD3-αCD28 targets HIV-1 and T cells and it was detected in high levels in the peripheral and secondary lymph tissues. This resulted in the potent CD4+ and CD8+ T-cell activation against HIV-infected cells. T cells were particularly activated in follicular areas of the lymph nodes, where HIV reservoirs are known to reside. The trispecific Abs were also sufficient to redirect killing activity against Env-expressing target cells in the plasma [133]. This proof-of-concept for HIV-1 treatment is an adaption of a current clinical trial (NCT04401020) that has been designed for myeloma cells (i.e., lymphomas and leukemias) which is made of trispecific αCD38/αCD3-αCD28 [134]. 

For there to long-term control of HIV, immunotherapy poses the most promising to educate the CD4^+^ and CD8^+^ T cells that can coordinate responses involved in innate and adaptive immunity with an antigen-presenting cell. ART therapy could be enhanced with immunotherapy such as this trispecific N6/αCD3-αCD28 antibody, with the goal to eradicate HIV reservoirs. It is, however, imperative to recognize that an effective cure would confer protection against future infections.

### 4.9. ADCs as a Tailored Cancer Treatment

Antibody-drug conjugates (ADCs) represent a paradisgn shit in cancer therpay, marrying the precision of monoclonal antibodies with the potency of highly cytotoxinc agents. This integration holds promise in diminishing the severity of side effects by selectively directing their payload to the tumor site. Often likend to a “biological missile”, ADCs have become one of the fastest evolving classes of cancer therapeutics [135] due to the ability to strongly necessitate and stimulate the development the chemo-selective and biorthogonal chemistries for improved indices [136,137]. This stragegic incorporation of ADCs into multipfaceted cancer treatment regimens underscored their pivotal role in contemporary oncology [138].

### 4.10. bnAbs in Some Non-Cancer-Causing Viruses

Dengue virus (DENV) and Zika virus (ZIKV), without any direct link with cancer, can be treated with the use of monoclonal antibodies. Both DENV and ZIKV have dangerous consequences, including fatal hemorrhagic fever, microcephaly, and Guillain-Barré syndrome [139]. The two viruses are closely related, and are both transmitted in a similar fashion, through mosquito bites [139]. Structurally, DENV possesses an envelope I and non-structural protein 1 (NS1) that are important in subunit vaccines [140]. Similarly, ZIKV codes for 3 structural proteins, including an E protein, and several non-structural proteins in its viral RNA [141]. The E protein of each virus can span the length of the virus’ surface and, because of this, both quaternary and cryptic epitopes are of interest in the pursuit of neutralizing and monoclonal antibodies for DENV and ZIKV [142]. Most known neutralizing antibodies for Zika and Dengue block virion binding or target the E protein and prevent fusion [143].

ZIKV-117 and MZ4 are two neutralizing antibodies that both target the E protein of the Zika virus. ZIKV-117 corresponds to a quaternary epitope of the E protein dimer of the Zika virus, while MZ4 targets the D1/DIII linker region of the DENV [144,145]. Another antibody, 5J7, binds to a quaternary epitope, like ZIKV-117 does. This epitope includes a wide span of E protein, including the DI-DII hinge, DII, and DIII domains of DENV [142,146] (Table 2). There are also neutralizing antibodies that are both Zika and Dengue specific. One such antibody is EDE1-C8, that targets the E- dimer and provides neutralizing protection against Zika and Dengue (1–4) [147].

### 4.11. Challenges and Limitations of bnAb Therapy

Although pre-clinial models of passive bnAbs therapy for alternative HIV treatments or complement to ART has shown progress, factors like antibody decay, viral latent resevior presistnace, and resistance have presented unique challenges to implementing this therapeutic route [148]. Several studies have investigated and strategied options to address current challenges. Some of these approaches include increasing potentcy and half-life of bnAbs and introducing vector mediated gene expression strategies [98]. Additional approaches of combination antibody therapy and evolving our understanding of cell-to-cell transmission of the virus may promote its utility in preventing spread. Overall, given the difficulties in developing viral vaccines, bnAbs offer a promising strategy in suppressing viraemia and many candidates are progressing towards clinical stages. 

## 5. Conclusions and Future Perspectives

In summary, the intersection of chronic viral infections, particularly in PLWHA, and cancer poses significant challenges to healthcare. The intricate interplay between viral pathogens and the immune system underscores the need for multifaceted approaches to mitigate cancer risk in this population. Cutting-edge immunotherapies, such as DC-based vaccines, CAR-T cell therapies, and neutralizing monoclonal antibodies, show promising results in bolstering antiviral and anticancer responses. Furthermore, the development of broadly neutralizing antibodies tailored to specific viral infections, such as HTLV-1 and EBV, has provided a critical avenue for therapeutic intervention. These bnABs, targeting distinct epitopes, offer solutions for preventing and combatting viral infections associated with cancer. An integrated approach encompassing prevention, early detection, and advanced therapeutic modalities holds the key to reducing cancer risk in individuals affected by chronic viral infections. Vaccination against high-risk viruses like HPV and hepatitis stands as a cornerstone in this endeavor. Alongside vaccination, routine screening for both sexually transmitted infections and early signs of cancer is paramount to the early detection and management of potential malignancies. Moreover, lifestyle modifications can play a pivotal role in minimizing cancer risk. Engaging in safe sex practices and abstaining from behaviors associated with substance abuse can significantly contribute to this effort. Education and awareness campaigns aimed at promoting healthy lifestyles, especially within PLWHA communities, are essential in fostering a proactive approach toward cancer prevention. 

In tandem with these efforts, ongoing research into the development of targeted immunotherapies and the improvement of existing strategies will continue to drive progress in this field. Collaborative efforts between clinicians, immunologists, and virologists will be instrumental in translating these advancements into tangible benefits for patients. Additionally, investing in novel therapeutic targets and enhancing existing interventions will pave the way for more effective and personalized approaches to combat viral-associated cancers. In conclusion, the dynamic landscape of viral-associated cancers demands a multifaceted approach, incorporating preventative measures, early detection strategies, and innovative immunotherapies. Through concerted efforts and continued research endeavors, we can forge a path towards a healthier future for individuals affected by these complex health challenges.

## Figures and Tables

**Table 1:** Current and potential treatment strategies in virus-specific cancers.

Strategy	Infection	Associated Cancer	Target Protein	Approach Used
Immunotherapy	HCV	Liver cancer	Core_133–142_	Immunization with dendritic cells treated with an anthrax toxin fusion protein [45]
			Core, NS4, NS3	Generate HCV-specific CTL from I precursors with peptide-pulsed DCs [45]
			NS5	Immunization with dendritic cells containing NS5-protein-coated microparticles [46]
			NS3	Fusion protein incorporating the extra domain A (EDA) from fibronectin and the HCV NS3 [47]
			Core and NS3	Transfection of DCs with recombinant AV or AAV [47]
			HCV-like particles (HCV-LPs)	Ex vivo-generated DCs using ex vivo antigen [47]
	HIV-1	Kaposi’s Sarcoma, non-Hodgkin’s lymphoma, and cervical cancer	HIV-derived CD4^+^ T cell epitopes (HIVBr8)	Chimeric monoclonal antibodies directed to DC surface receptors fused to the antigen of interest [48]
			Tat and Vpx proteins	mRNA vaccine [48]
	EBV	Nasopharyngeal carcinoma	Nuclear antigen EBNA1 Membrane protein LMP2	Dual stimulation of virus-specific CD4+ and CD8+ T-cell responses by a chimeric antigen construct that contains two viral fusion epitopes [49]
	HTLV	T-cell leukemia	Tax_11–19_	DC-based anti-HTLV-1 vaccine [50]
				Autologous dendritic cell vaccine pulsed with class I peptides from tumor-associated antigens [51]
NK Cell Therapy	HIV			Dual blockade of PD-1 and IL-10 in NK cells [52]
Chimeric Antigen Receptor T-Cell			Envelope protein gp120	Autologous CD4+ and CD8+ T cells transduced with a lentiviral vector encoding bispecific anti-gp120 CAR molecules (LVgp120duoCAR-T) [53]

## Data Availability

No new data were created or analyzed in this study. Data sharing is not applicable to this article.
